# Assessment of the Geographic Origins of Pinewood Nematode Isolates via Single Nucleotide Polymorphism in Effector Genes

**DOI:** 10.1371/journal.pone.0083542

**Published:** 2013-12-31

**Authors:** Joana Figueiredo, Maria José Simões, Paula Gomes, Cristina Barroso, Diogo Pinho, Luci Conceição, Luís Fonseca, Isabel Abrantes, Miguel Pinheiro, Conceição Egas

**Affiliations:** 1 Department of Life Sciences, University of Coimbra, Coimbra, Portugal; 2 Genoinseq, Next Generation Sequencing Unit, Biocant, Cantanhede, Portugal; 3 IMAR-CMA, Department of Life Sciences, University of Coimbra, Coimbra, Portugal; Pennsylvania State University, United States of America

## Abstract

The pinewood nematode, *Bursaphelenchus xylophilus*, is native to North America but it only causes damaging pine wilt disease in those regions of the world where it has been introduced. The accurate detection of the species and its dispersal routes are thus essential to define effective control measures. The main goals of this study were to analyse the genetic diversity among *B. xylophilus* isolates from different geographic locations and identify single nucleotide polymorphism (SNPs) markers for geographic origin, through a comparative transcriptomic approach. The transcriptomes of seven *B. xylophilus* isolates, from Continental Portugal (4), China (1), Japan (1) and USA (1), were sequenced in the next generation platform Roche 454. Analysis of effector gene transcripts revealed inter-isolate nucleotide diversity that was validated by Sanger sequencing in the genomic DNA of the seven isolates and eight additional isolates from different geographic locations: Madeira Island (2), China (1), USA (1), Japan (2) and South Korea (2). The analysis identified 136 polymorphic positions in 10 effector transcripts. Pairwise comparison of the 136 SNPs through Neighbor-Joining and the Maximum Likelihood methods and 5-mer frequency analysis with the alignment-independent bilinear multivariate modelling approach correlated the SNPs with the isolates geographic origin. Furthermore, the SNP analysis indicated a closer proximity of the Portuguese isolates to the Korean and Chinese isolates than to the Japanese or American isolates. Each geographic cluster carried exclusive alleles that can be used as SNP markers for *B. xylophilus* isolate identification.

## Introduction

*Bursaphelenchus xylophilus*, the pinewood nematode (PWN), is a migratory plant-parasite nematode and the causal agent of the pine wilt disease. This nematode is transmitted from tree to tree by insect vectors, mainly belonging to the genus *Monochamus* [1]–[3]. The nematodes enter pines through the wounds caused by the insect feeding on the twig bark. Once inside the tree, they feed and reproduce along the resin canals of xylem and cortex, leading to cambial necrosis and tree death within months [4]–[6]. First detected in North America (USA and Canada), the PWN spread to Japan in the early part of the twentieth century. In North America, the nematode caused limited damage, however, it became a severe conifer pest in Japan [7]. In the 1980s, it spread to other Asian countries like China and Korea and was detected for the first time in Europe, in 1999, in the Setúbal Peninsula, Continental Portugal [8]. In 2008, the PWN spread to new forest areas in the Portuguese centre region. Currently, the nematode is also present in Madeira Islands [9] and Spain [10]. *B. xylophilus* is a quarantine pest of the European and Mediterranean Plant Protection Organization (EPPO) since 1986 [11]. The EU Commission decision 2006/133/CE imposed strict measures to prevent nematode spread to neighboring regions, imparting a heavy economic burden on affected countries. The accurate detection of the species and its dispersal routes are thus essential to define effective control measures [9].

While the biology of the PWN is well understood [12], the molecular mechanism of *B. xylophilus* pathogenicity remains to be elucidated. Stylet secretions delivered into the host are expected to play a major role in the disease, with proteins that soften or degrade plant cell walls, suppress or avoid host defenses and manipulate host signaling pathways [13], [14]. These proteins, named effectors, have been described for several nematode species [13]–[16] and some of the already known genes have also been found in *B. xylophilus* through analysis of expressed sequence tags (EST) [17] and genome sequence [18]. PWN produces a battery of cell wall-degrading enzymes, including cellulases, pectin lyases and other proteins involved in cell wall molecule interactions such as expansin, that helps the nematode to invade and migrate within plant tissues [14]. Beta-1,4-endoglucanase degrades cellulose, the main component of the plant cell wall, pectate lyase cleaves pectate internal alpha-1,4 linkages and expansin disrupts non-covalent bonds between polysaccharide chains, making them more accessible to hydrolytic enzymes. These enzymes are produced in oesophageal glands and secreted via the stylet [19]–[21]. Immunologic studies additionally identified cellulases in the tracheid cells and resin canals of the infected plant tissues [22]. Another glycosyl hydrolase, beta-1,3-endoglucanase has been found in the EST study [17]. This enzyme degrades components of the fungal cell wall and is produced when the PWN is feeding on fungi. The role of chitinase on the molecular mechanisms of the pine wilt disease is not clear. The identification of a chitinase-like gene in *Pratylenchus coffeae*, a non-fungal feeding nematode, suggests a role in the degradation of other substrates such as callose, a polymer of beta-1,3-glucan that is deposited by plants under stress conditions [23].

Venom allergen-like proteins (VAP) have also been described as important secreted effector proteins. These proteins induce host immune responses in animal parasites and are conserved in several species of plant parasite nematodes [13]. Cysteine-rich VAPs were found in the oesophageal glands of the PWN, although its function in parasitism has not yet been clarified [18], [24]. Another class of effector proteins has potential functions in the proteasome, suggesting that nematodes may actively regulate host cell protein degradation, most probably to suppress host defense [15], namely proteins similar to SKP-1 and RING [13], [25]. SKP-1 (S-phase kinase associated protein) is a key component of the SCF (Skp-Cullin-F-box) E3 complex that is involved in a variety of signal transduction pathways [26]. Some RING proteins are involved in the transfer of ubiquitin to proteins targeted for subsequent degradation within the cell [26]. In *Heterodera glycines*, RING H2 is involved in the disruption of SA-mediated defense signaling [27]. Although SKP-1 and RING proteins are present in the PWN [17], their secretion by oesophageal glands has not yet been experimentally proven.

Single nucleotide polymorphisms (SNPs) are point alterations of alleles at a locus, and can be used to detect differences among individuals of a species. SNPs have relatively low mutation rates and provide co-dominant data [28]. Those located in coding regions additionally differentiate loci under selective pressure from neutral loci [29], and can provide information on functional diversity among individuals or isolates. Restriction fragment length polymorphisms (RFLP) or simple sequence repeats (SSR) are equally powerful markers, however, SNP markers come from sequence information and are highly reproducible [31]. Furthermore, SNP genotyping is currently an accurate, scalable, cost-effective process for the simultaneous detection of hundreds of polymorphisms [30], [32], [33].

SNP genotyping is widely used in well studied organisms such as humans [34] or model plants [35], [36], for which genomes or genomic information already exist. For non-model organisms, EST analysis has been a resourceful data set [37], [38]. Recent advances in sequencing technology and bioinformatics now offer the opportunity to generate information on genome-wide EST collections, or transcriptomes, in a high throughput low cost manner [39]–[41]. Although the main applications of transcriptome sequencing have been gene discovery and expression profiling [42]–[44], comparative transcriptome analysis of different individuals or cells identifies common genes and uncovers polymorphisms. This approach has been applied to address genetic diversity and identify genetic markers in several species such as oat [45], melon [46] or insects [47], but there are few reports of its use in nematodes [48], [49].

To sequence a transcriptome, cDNA is synthesized from mRNA of an individual or pool of individuals and then sequenced with a second generation sequencing technology. In cases where the genome sequence is still unavailable, the Roche 454 pyrosequencing technology [50] has been a preferred platform for its longer read lengths [51]. In this technology, the cDNA from an individual or pool of individuals is amplified with emulsion polymerase chain reaction and pyrosequenced in a high throughput parallelized process that provides equal opportunity for all molecules in the pool to be sequenced at high coverage. Transcripts comparative analysis between different individuals facilitates the detection of high quality polymorphic positions, thus providing a valuable data set to retrieve information on genetic diversity.

In this study, we analyse the genetic diversity among PWN isolates from different geographic locations and identify SNPs in putative effector genes that can be used as markers for the origin of *B. xylophilus* isolates, through a comparative transcriptomic approach using the high throughput 454 sequencing platform.

## Materials and Methods

### Nematode isolates

*Bursaphelenchus xylophilus* isolates from different geographical origins (Tables 1 and S1 in File S1) were selected from the pinewood nematode collection of the Laboratory of Nematology, IMAR-CMA, UC, established and maintained in cultures of *Botrytis cinerea*, grown on malt extract agar medium, and incubated at 25°C [52].

**Table 1:** *Bursaphelenchus xylophilus* (Bx) isolates and their respective geographic origins (Pt – Continental Portugal, J – Japan, Ch- China and USA - United States of America).

Isolate code	Geographic origin	*Pinus* species
BxPt15SC	Santiago do Cacém (Setúbal district, Continental Portugal, initial outbreak)	*P. pinaster*
BxPt17AS	Alcácer do Sal (Setúbal district, Continental Portugal, initial outbreak)	*P. pinaster*
BxPt19SCD	S. Comba Dão (Coimbra district, Continental Portugal, 2008 outbreak)	*P. pinaster*
BxPt21T	Tábua (Coimbra district, Continental Portugal, 2008 outbreak)	*P. pinaster*
BxJ10	Japan (Fukushima, Pref.Nishiaizu)	*P. densiflora*
BxChJS	China (Jiangsu province)	Unknown
BxUSA618	USA (unknown)	Unknown

### RNA extraction, cDNA library construction and pyrosequencing

Nematodes (mixed developmental stages) from seven isolates (Table 1) were collected from fungal cultures, washed in sterile water for contaminant removal and immediately frozen in liquid nitrogen. Aliquots of ca. 15,000 nematodes from each isolate were separately ground in liquid nitrogen using a mortar and pestle until powder and homogenized 20 times through a 20 gauge syringe needle [53]. The homogenates were processed for total RNA extraction with Trizol, according to standard manufacturers' instructions (Invitrogen, Carlsbad, CA). The quality was verified on Agilent 2100 Bioanalyzer (Agilent Technologies, Palo Alto, CA) with the RNA 6000 Pico kit (Agilent Technologies, Palo Alto, CA) and the quantity assessed by fluorimetry with the Quant-iT RiboGreen RNA kit (Invitrogen, CA, USA).

A fraction of 1–2 micrograms for each isolate was used as starting material for cDNA synthesis with the MINT cDNA synthesis kit (Evrogen, Moscow, Russia) to amplify selectively mRNA through polyA tails, using a modified template-switching approach that allows the introduction of known adapter sequences to both ends of the first-strand cDNA. The cDNA library was then normalized according to the Duplex-Specific Nuclease-technology [54], following the instructions of the TRIMMER cDNA Normalization kit (Evrogen, Moscow, Russia).

The cDNA libraries were fragmented by nebulization with N_2_ and the fragments ligated to sequencing adaptors containing MID barcodes for sample identification, according to the 454 GS FLX standard protocol (Roche-454 Life Sciences, Brandford, CT, USA). The seven ssDNA libraries were quantified by fluorescence with the Quant-iT RiboGreen RNA kit (Invitrogen, CA, USA), pooled in equimolar amounts and pyrosequenced at Biocant (Biocant, Cantanhede, Portugal) in a single plate with GS FLX (Roche-454 Life Sciences, Brandford, CT, USA), according the standard manufacturers' procedures.

### Transcript clustering, SNP calling and functional annotation

Following 454 sequencing, the reads were trimmed for quality and selected according to size by the 454 software (Roche-454 Life Sciences), and the MINT adaptor sequences removed from reads using a custom script. The reads were then submitted to MIRA (version 3.0.5) [30] for clustering and SNP calling, under default parameters. The MIRA application is an assembler for mRNA reconstruction, however, it can cluster reads instead. This clustering option assembles transcripts into one consensus sequence, allowing for polymorphic position identification [30]. The translation frame of the transcripts was determined through queries against the NCBI non redundant protein database using BLASTx [55] with an E-value of 10^−6^ and assessing the best twenty five hits. Contigs without hits were submitted again to BLASTx homology searches against the NCBI nr database with a higher E-value cut-off set at 10^−2^. Sequences with an identified translation frame from the two previous searches were used to establish the preferential codon usage in *B. xylophilus* based on which the software ESTScan [56] further detected putative transcripts on sequences with yet no BLASTx matches. This procedure originated a third set of sequences with putative amino acid translation. The entire collection of sequences with at least 30 amino acids long, resulting from the BLASTx and the ESTScan procedures, was processed by InterProScan for protein domain signatures prediction. The entire reads set used for the final assembly was submitted to GenBank, in the Sequence Read Archive under the accession n° SRA068546.

### Validation of SNPs in Genomic DNA: Genomic DNA isolation, Sanger sequencing and chromatogram analysis

Nematodes (mixed developmental stages) from 15 PWN isolates, the seven pyrosequenced isolates plus eight from Madeira Island, China, Japan, USA and South Korea (Table S1 in File S1), were collected from fungal cultures, washed in sterile water for contaminant removal and ground to powder in liquid nitrogen. The DNA was then isolated with the DNeasy Blood and Tissue kit (Qiagen, Valencia, CA, USA) according to the manufacturers' instructions. DNA quantity was measured in NanoDrop 1000 Spectrophotometer (Thermo Scientific, Delaware, USA) and the quality verified by agarose gel electrophoresis. Primers were designed with the software OligoExplorer (Table S2 in File S1) and used to amplify each effector gene from genomic DNA for SNP validation. The amplification reactions were performed in a final volume of 50 µl containing 100 ng of DNA, lx reaction buffer, 2 mM MgSO_4_, 0.2 mM dNTPs, 0.2 µM of each primer and 2U *Taq* Platinum High Fidelity Taq (Invitrogen, Life Technologies, Carlsbad, CA). All the amplifications were carried out in a MyCycle Thermal Cycler (Bio-Rad, California, USA) with the following PCR program: initial denaturation step at 94°C for 3 min, followed by 30 cycles of denaturation at 94°C for 30 s, annealing for 30 s, with the temperature adjusted for each primer pair, extension at 68°C for 1 min, and a final extension step at 68°C for 2 min. The amplified fragments were purified using the High Pure PCR Product Purification Kit (Roche Applied Science, Penzberg, Germany) according the standard manufacturers procedures. Each amplicon was sequenced in forward and reverse directions by Sanger standard procedures at Biocant (Biocant, Cantanhede, Portugal). Sequencing chromatograms were aligned and the polymorphisms analysed using BioEdit [57].

### Analysis of polymorphisms and isolate clustering

Major Allele Frequency (MAF) was determined with the software SNPAnalyzer-2™ [58], using the online website (http://snp.istech21.com/snpanalyzer/2.0/). The effect of amino acid changes in the proteins encoded by the transcripts was predicted in the online SIFT platform (http://sift.jcvi.org/) using the SIFT Sequence option [59].

Evolutionary divergence (p-distance) between nucleotide sequences was computed from pairwise analysis using the Maximum Composite Likelihood method [60] implemented in MEGA V5.0 package [61]. Isolate clustering was addressed through the Neighbor-joining approach [62], based on pairwise distance matrices between samples, and Maximum Likelihood method [60] computed using the Jukes-Cantor model [63]. The Neighbor-joining and Maximum Likelihood trees were constructed using functions in the MEGA V5.0.

Isolate clustering was additionally determined using the alignment-independent bilinear multivariate modelling (AIBIMM) approach [64]. For this analysis, the DNA sequences were transformed into a DNA pentamer frequency table using the computer program PhyloMode (http://www.nofimamat.no/phylomode). A window of five nucleotides was moved along each DNA sequence and the frequencies of different pentamers present in the sequence were stored in a frequency table. The pentamer frequency table was then compressed using principal component analysis, (PCA), a projection method that transforms a data table consisting of possibly correlated variables into a smaller number of uncorrelated latent variables, that reflect the most important structure of the data [65].

## Results

### Sequencing, transcript clustering and annotation

The total RNA of *B. xylophilus* isolates from Continental Portugal (2 from the initial outbreak, 2 from the 2008 outbreak), USA (1), China (1) and Japan (1) (Table 1) was extracted and used to prepare normalized cDNA libraries for each isolate. The seven libraries were pyrosequenced in a single GS FLX plate, generating 597,055 raw nucleotide reads of a medium length of 228 bp (Table 2). In order to detect SNPs among the PWN isolates, the software MIRA SNPs [30] clustered the reads of the seven isolates together, creating 21,566 transcripts of an average length of 552 bp (Table 2). Annotation of the transcripts by queries against the InterPro database of protein families and functional domains [66] identified 17,772 hits. Almost half of the transcripts had best BLAST hits within nematodes. Most of these matched *Caenorhabditis elegans*, 5,142 contigs, and *C. briggsae*, 2,129 transcripts, while 3,388 transcripts matched *Brugia malayi*. Only 59 transcripts matched *Bursaphelenchus xylophilus*, probably reflecting the lack of annotated genes in public databases.

**Table 2:** Summary of sequencing, clustering and annotation results.

Parameter	Results
Number of reads	597,055
Total bases (bp)	138,271,100
Average read length (bp)	228
Number of transcripts	21,566
Average contig length (bp)	552
Range contig length (bp)	84–8,800
Contigs with *BLASTx matches (E-value≤10^−6^)*	13,261
Contigs translated with ESTscan	8,305
Total number of translated amino acid sequences	27,633
Transcripts with InterPro annotation	17,772

The reads from all libraries were clustered with the software MIRA SNPs and annotated according to conserved protein domains in InterPro.

### Identification of SNPs in putative effector genes

The genetic diversity among the isolates from different geographic locations was addressed by studying a defined set of known genes. For this, a literature search identified putative effector and parasitism genes as potential candidates [15], [16], [18], namely cellulase, expansin and pectate lyase, involved in plant cell wall degradation. Other candidate genes identified were venom-allergen proteins (VAPs), calreticulin and annexin, predicted to modulate host defence or the cell cycle. Additional targets were ubiquitin, SKP1 and RING genes, involved in the ubiquitination process, and lastly chitinases, that degrade the fungal cell wall. Manual curation of clustered reads for each transcript, in a total of 87 transcripts, identified nucleotide diversity in 26 transcripts, corresponding to eight genes (Table 3). The process eliminated SNPs in read extremities, known to have a higher error rate, SNPs next to homopolymers and those positioned in regions where local coverage was below three reads from distinct PWN isolates. This analysis revealed 155 putative SNPs (Table 3).

**Table 3:** Genes selected for single nucleotide polymorphism (SNP) evaluation and their predicted function in parasitic nematodes.

Gene	Transcripts #	Function	Category
Beta-1,4-endoglucanase	3	Hydrolysis of beta-1,4-glucan [19]	
Expansin	2	Cell wall extension [21]	Plant cell wall degradation [16]
Pectate lyase	1	Hydrolysis of pectate alpha-1,4-linked galacturonic acid [20]	
Venom-allergen protein	1	Unknown [18]	Unknown [53]
Ubiquitin	1	Selective protein degradation [15]	
SKP1	1	Involved in signal transduction [15]	Ubiquitination [53]
RING	13	Indirect induction of antioxidant genes in syncytium [15]	
Beta-1,3-endoglucanase	4	Egg hatching/fungal feeding [17]	Unknown [53]

Identified SNPs were validated in genomic DNA as the number of SNPs identified by transcriptome sequencing is usually inflated, most probably as a consequence of a higher error rate of reverse transcriptase as compared to DNA polymerases. Indeed, confirmation of a few SNPs by Sanger sequencing from cDNA and then from genomic DNA showed a few false positive cDNA polymorphisms (results not shown). Validation involved the amplification of the six genes by designing primers according to the transcript sequence, followed by Sanger sequencing. The SNPs previously identified in VAP (1 SNP) and in Ubiquitin (2 SNPs) could not be confirmed. The validation included eight additional PWN isolates from Madeira Island (2), Portugal, China (1), United States (1), Japan (2) and finally South Korea (2) (Table S1 in File S1). All transcripts amplified from genomic DNA, thus validating the correctness of the clustering process. Additionally, the specificity of primers was validated by amplification of genomic DNA from *B. mucronatus*, a non-pathogenic close species [52], without success. Sanger sequencing confirmed 104 SNPs identified by MIRA and further detected 32 new polymorphisms that were not correctly called due to low coverage. The validation step identified 136 polymorphic positions in 10 transcripts corresponding to 6 different genes (Tables 4 and S1 in File S1).

**Table 4:** Transcripts of *Bursaphelenchus xylophilus* sequenced for genotype validation, number of identified single nucleotide polymorphisms (SNPs) and InterPro categories.

Gene	Transcript name	Size (bp)	# SNP	InterPro ID	InterPro description
Cellulase	c4171	615	22	IPR000334	Glycoside hydrolase, family 45
	c7206	544	12		
	c1471	778	23		
Chitinase	c4666	876	1	IPR001223	Glycoside hydrolase, family 18, catalytic domain
	c9244	629	24		
Expansin	c5646	386	12	IPR009009	Barwin-related endoglucanase
Pectate lyase	c5837	484	1	IPR004898	Pectate lyase, catalytic
SKP1	c4717	538	19	IPR001232	SKP1 component
RING	c2443	685	17	IPR001841	Zinc finger, RING-type
	c6704	223	5	IPR000571	Zinc finger, CCCH-type

### Analysis of SNPs detected in *B. xylophilus* isolates

The sequence of the ten transcripts corresponded to 5,758 bases, yielding a frequency of 2.4% polymorphic positions. These SNPs corresponded only to variation in exons. SNPs were also detected in introns; however, these were not considered for analysis due to large variability found in intron size and composition between isolates from the different geographic regions. The majority of the SNPs were homozygous in each isolate (74%). Remaining SNPs where heterozygous but they were only observed in this state in the two isolates from USA (BxUSA618, BxUSA745) and in one of the Japanese isolates (BxJT4) (Table S3 in File S1). The major allele frequency was estimated for each SNP as a measure of SNP variability [46]. The proportion of SNPs with a major allele frequency <0.9 was 82%, however, the percentage of highly variable SNPs, with MAF<0.7, was low (18%) (Table S4 in File S1). Furthermore, the low heterozygosity observed, and concomitant deviation from the Hardy Weinberg equilibrium, prevented the characterization of population genetic parameters for the polymorphic positions.

Among the 136 SNPs, only 5 polymorphisms corresponded to non-synonymous alterations. Three non-synonymous alterations were G to A transitions, responsible for the amino acid change of a valine for an isoleucine in the SKP1 transcript (c4717), a glycine to serine change in the chitinase c9244 and a valine into a methionine in the RING transcript (c2443) (Table S5 in File S1). In SKP1 transcript, there was a transition of a C to T, changing a threonine into a methionine. The fifth non-synonymous alteration corresponded to a double change of the first nucleotide of the codon, a transition of A per G, and the third codon nucleotide, a transversion from C to G, changing an asparagin to a glutamic acid (Table S5 in File S1). The evaluation of the effect of amino acid changes in the protein with SIFT [59] indicated all amino acids as tolerated.

### Clustering of *B. xylophilus* isolates according to SNP diversity

The 136 polymorphic positions in each transcript were concatenated into a single sequence, and one sequence generated for each isolate. These 136 bp synthetic sequences were utilized as input for further analysis. The genetic distance among isolates was calculated as pairwise distance (p-distance) through matrix calculations (Table 5). There were no differences between Portuguese isolates from the initial outbreak and the isolates from Madeira Island, whereas the Portuguese isolates from the later outbreak differed in four nucleotides in RING c6704 (p-distance of 0.037). Taking the Portuguese PWN isolates as the reference, the p-distance was 0.257 for the Chinese isolates, between 0.310–0.647 for the Japanese isolates and 0.430–0.540 for the USA. The South Korean isolates were identical to the Portuguese isolates from the first outbreak (BxPt15SC, BxPt17AS) and the isolates from Madeira Island (BxMad4SV, BxMad16S).

**Table 5:** Pairwise distance among *Bursaphelenchus xylophilus* (Bx) isolates.

Isolate code	BxPt15SC	BxPt17AS	BxMad4SV	BxMad16S	BxKAS	BxKBG	BxPt19SCD	BxPt21T	BxChJS	BxChSD	BxJ10	BxJT4	BxJS10	BxUSA618	BxUSA745
**BxPt15SC**															
**BxPt17AS**	0.000														
**BxMad4SV**	0.000	0.000													
**BxMad16S**	0.000	0.000	0.000												
**BxKAS**	0.000	0.000	0.000	0.000											
**BxKBG**	0.000	0.000	0.000	0.000	0.000										
**BxPt19SCD**	0.037	0.037	0.037	0.037	0.037	0.037									
**BxPt21T**	0.037	0.037	0.037	0.037	0.037	0.037	0.000								
**BxChJS**	0.257	0.257	0.257	0.257	0.257	0.257	0.265	0.265							
**BxChSD**	0.257	0.257	0.257	0.257	0.257	0.257	0.265	0.265	0.000						
**BxJ10**	0.632	0.632	0.632	0.632	0.632	0.632	0.640	0.640	0.640	0.640					
**BxJT4**	0.310	0.310	0.310	0.310	0.310	0.310	0.317	0.317	0.317	0.317	0.325				
**BxJS10**	0.647	0.647	0.647	0.647	0.647	0.647	0.625	0.625	0.654	0.654	0.015	0.341			
**BxUSA618**	0.540	0.540	0.540	0.540	0.540	0.540	0.540	0.540	0.613	0.613	0.540	0.579	0.540		
**BxUSA745**	0.430	0.430	0.430	0.430	0.430	0.430	0.405	0.405	0.471	0.471	0.430	0.393	0.413	0.303	

The distances were calculated based on 136 positions for the 15 isolates in MEGA5.

**Figure 1:**
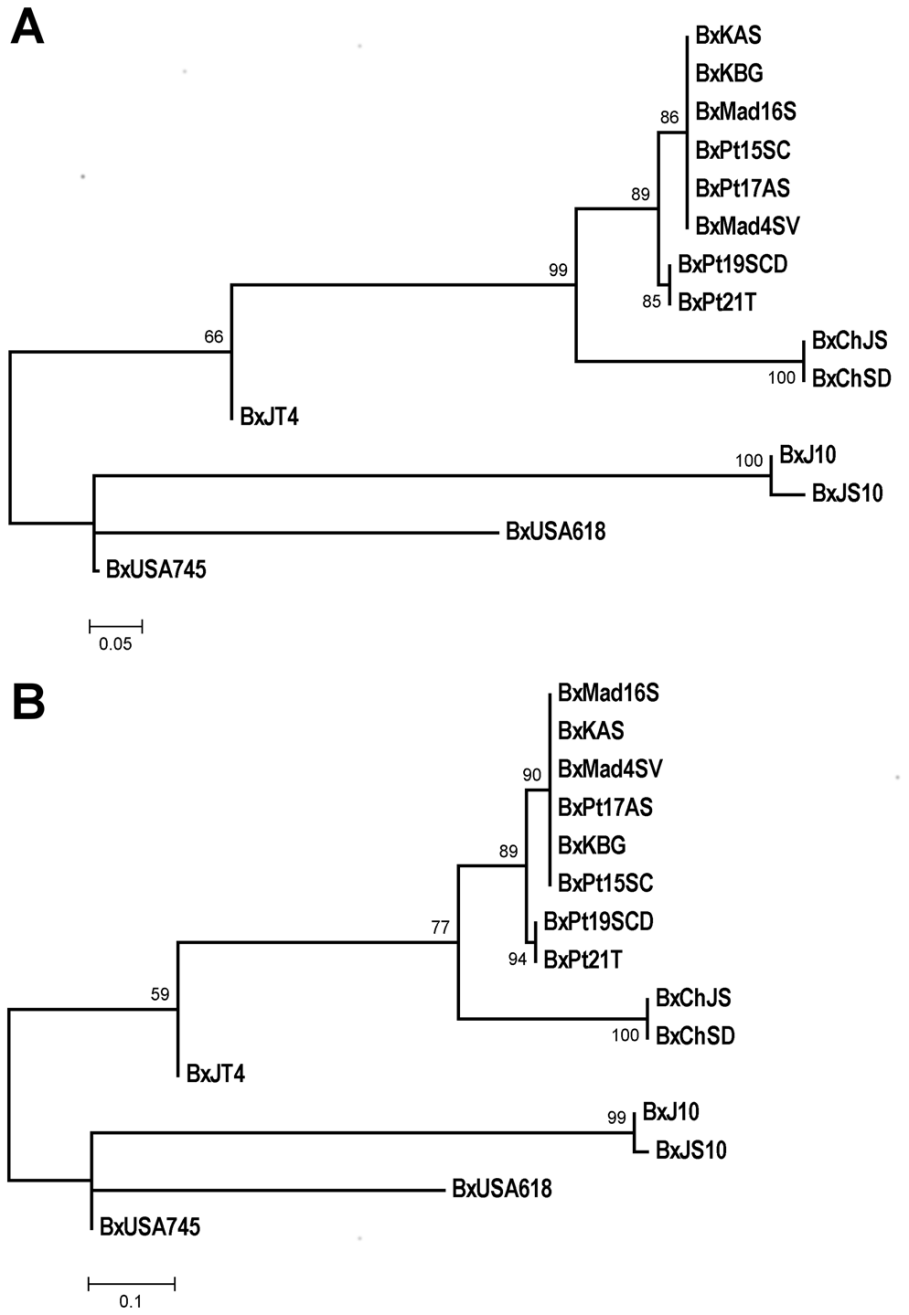
Phylogenetic trees based on the single nucleotide polymorphism (SNP) of 10 transcripts in 15 *Bursaphelenchus xylophilus* (Bx) isolates from different geographic locations. The tree was based in the *Neighbor-Joining* algorithm (A) and Maximum Likelihood (B) and the nodes supported by 1000 bootstrap repetitions. The trees were produced with MEGA 5.

PWN isolates clustering was analysed with Neighbor-joining [62] and Maximum Likelihood methods [60] using the synthetic sequences. Results from the two approaches were congruent and distributed the isolates according to their geographic origins (Fig. 1). The Portuguese isolates from the initial outbreak, the isolates from Madeira Island and the Korean isolates clustered in the same clade. The two Portuguese isolates BxPt19SCD and BxPt21T, from the central region of the country, clustered together but in a sister clade. The two isolates from China clustered together and formed a sister clade to the Portuguese isolates with a high bootstrap node value (99% for Neighbor-joining and 77% for Maximum Likelihood). The Japanese isolate, BxJT4, formed its own phylogenetic group, close to the Portuguese, Korean and Chinese isolates. The other two Japanese isolates (BxJ10, BxJS10) and the two USA isolates clustered in a different node. The Japanese isolates clustered together in a clade, and the two American isolates positioned in independent branches. No outgroups were considered in this analysis due to the lack of amplification of *B. mucronatus* with the gene specific primers for PWN and the lack of sequence information for the gene set addressed in this study.

**Figure 2:**
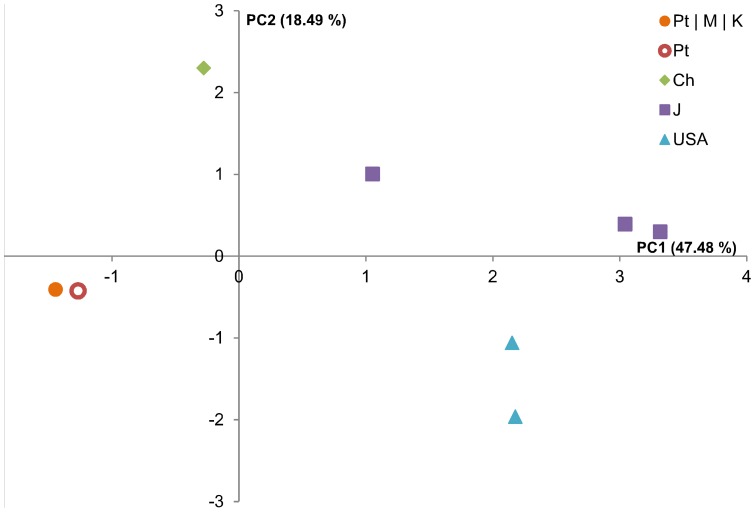
Principal component analysis of the 5-mer frequency in the 136 nucleotide sequences of the 15 *Bursaphelenchus xylophilus* isolates (Bx), determined by the alignment-independent bi-linear multivariate modelling (AIBIMM) approach. The legend on each axis indicates the variances (%) for principal components 1 and 2. The group Pt/M/K (closed orange circle) represents the Portuguese isolates from the initial outbreak, BxPt15SC, BxPt17AS, M - isolates from Madeira Island, BxMad4SV, BxMad16S, and K the two Korean isolates, BxKBG and BxKAS. Pt (open orange circle) represents the isolates from the 2008 outbreak in the central region of Portugal, BxPt19SCD and BxPt21T. Ch (green diamond) represents Chinese isolates BxChJS and BxChSD; J (purple squares) represents the Japanese isolates BxJ10, BxJS10, BxJT4, and finally USA (light blue triangles) represents BXUSA618 and BxUSA745.

PWN isolate clustering was further analysed using the alignment-independent bi-linear multivariate modelling (AIBIMM) approach [64]. This method transforms DNA sequences into an n-mer frequency table. After normalization, the frequency table is compressed using PCA, thereby transforming possibly correlated variables into a smaller number of uncorrelated variables, which reflect the most important structure of the data [65]. For this analysis, the complete sequences of the ten transcripts were concatenated in the same order for each isolate and each 5,758 base sequence analysed through a 5-mer window. The results, compiled in a frequency table, were compressed by PCA. The first three axes of PCA, PC1, PC2 and PC3, accounted for 80.31% of the total variance; the first axis, PC1 accounted for 47.48% of the total variance, PC2 for 18.49% and PC3 for 14.34%. The PWN isolates clustered according to their geographical origin (Fig. 2); the Portuguese isolates clustered together and with the South Korean. The two Chinese isolates formed a distinct cluster. The BxJT4 isolate positioned close to the other two Japanese isolates in the same quadrant and the American formed a distinct group.

### Detection of discriminating SNPs between the geographic PWN clusters

Analysis of SNPs between the clusters identified polymorphic positions in six transcripts that could differentiate PWN isolates according to the geographic clusters (Table S6 in File S1). The Portuguese and the two South Korean isolates had a set of seven SNPs in chitinase c9244 distinct from remaining clusters. The Chinese isolates could be distinguished by nine polymorphic positions in RING c2443, one in chitinase c9244 and one in expansin c5646. The Japanese isolates differed from other isolates in five SNPs in cellulase c7206, one in chitinase c9244 and one in SKP c4717. The USA isolates could be differentiated by two SNPs in cellulase c4171 and one SNP in SKP1 c4717.

## Discussion

The transcriptome sequencing of *B. xylophilus* isolates from four distinct geographic regions revealed inter-isolate variation in the coding regions of several genes. These were further analysed to discover SNP markers that correlate with geographic origin.

Indeed, the comparison of nematode putative effectors gene sequences highlighted 136 SNPs and a positive correlation with the nematode origin. Genetic diversity analysis showed that the Portuguese isolates were identical to those of South Korea, close to the Chinese isolates and distant from the Japanese and USA. Interestingly, the Korean isolates studied shared the same polymorphic positions with the Portuguese isolates.

Comparative transcriptome analysis is now a widely used tool to analyse molecular and functional information from non-model organisms [38], [51]. In the nematode field, this approach has, for example, provided massive amounts of information allowing to identify up-regulated genes involved in detoxification and protein degradation in the early parasitism stages of *Meloidogyne incognita* [67], differential gene expression between males and females of *Ancylostoma caninum* [42], functional characterization of plant parasitic nematodes such as *Pratylenchus thornei* [68], the migratory plant-parasitic peanut pod nematode *Ditylenchus africanus* [69], the animal parasitic nematode *Necator americanus*, the blood-feeding hookworm [70] or the heartworm *Dirofilaria immitis* [71]. The transcriptome sequencing of *B. xylophilus* also provided a functional overview of this nematode while growing on fungi. In this food-rich, non-parasitic environment, most of the expressed genes were similar to that of soil free-living *C. elegans*, although the two nematodes belong to different clades; *B. xylophilus* belongs to Clade 10 whereas *C. elegans* is included in Clade 9 [72], [73]. The genes shared between the two species may thus reflect genes participating in core mechanisms common to all nematodes. The relatedness of the two nematode genes had already been reported in the genome analysis of the PWN [18], and this study confirms the same profile in genes being expressed. A second group of genes had best hits in *Brugia malayi*, a human parasite responsible for lymphatic filariasis, additionally confirming the information provided by the PWN genome information [18].

While growing on fungi, PWN expressed genes involved in the cell wall-degrading enzymes such as beta 1–4 endoglucanases, pectate lyases and expansins [18]. The role of these genes in the feeding process on fungi is difficult to explain as these are not directly involved in the degradation of the fungal cell wall. This is not a new observation, though. Cellulases were detected in *Pristionchus pacificus* growing on bacterial lawns [74], and no evident role could be suggested. Conversely, PWN expressed beta-1-3 endoglucanase, probably related to the digestion of chitin, the major constituent of the fungal cell wall [75]. The nematode was also expressing other parasitism genes similar to those of parasitic plant nematodes, such as VAPs, or genes involved in targeted protein degradation like ubiquitin, RING and SKP1 [15], [16], [18]. In fact, the detection of genes putatively involved in parasitic mechanisms in the fungal culture of PWN suggests these genes may play different roles in the nematode and that other genes may be implicated in the molecular parasitism. Results from the comparative analysis of human, animal and plant parasitic nematode mechanisms from the gene/genome point of view highlighted the lack of an universal parasitic mechanism [76] and *B. xylophilus* may well use different genes or pathways to overcome the pine anti-nematodal response [77].

The genetic diversity of PWN using SNPs is for the first time addressed, the majority of the SNPs being homozygous. Heterozigosity was restricted to 35 out of 136 loci in the USA isolates and one of the Japanese isolates (BxJT4). Therefore, the genetic diversity of the loci among the isolates collected at different geographic locations was addressed by pairwise comparisons through the Neighbor-Joining method, the Maximum Likelihood method and AIBIMM, an alignment independent method. Results from these methods were congruent in resolving the fifteen PWN isolates into four groups. One of the clusters comprised the Portuguese and the South Korean isolates, the second involved the Chinese isolates. The third grouped the Japanese and the fourth the USA isolates.

The Portuguese cluster was fairly homogeneous, with only four loci in the same transcript encoding a RING protein discriminating the isolates from the initial outbreak and Madeira Island isolates from those collected after 2008. Variability studies within regions also reported low genetic variability among isolates, such as those of three Japanese forests [78], Chinese regions [79], [80] or Portuguese isolates [9], [81], [82]. However, inter-simple sequence repeats (ISSR) analysis showed high genetic variability among Portuguese isolates, except for the ones obtained prior to 2008 hypothesizing multiple introductions from different origins [81]. Further work with larger sample number and using other molecular methodologies will be necessary to confirm or exclude this hypothesis.

In the present study, the clustering of isolates showed that the Portuguese isolates were close to the Korean and the Chinese isolates and more distant to the Japanese and American isolates. An East Asian origin of the Portuguese isolates had already been described using ISSR/RAPD methodologies [83], sequence data of ITS [9], [59] and IGS [84] regions, cellulase genes [85] and DNA mitochondrial genes [82], [85]. The two South Korean isolates grouped together with the Portuguese isolates from the initial outbreak, sharing exactly the same alleles as the Portuguese isolates. Regarding the high diversity, reported for the South Korean isolates [86], it may have happened that the isolates available for this study originated in the same Korean region, and thus did not group differently. The results obtained with SNP markers for American and Japanese isolates were congruent with clusters obtained with other molecular markers such as AFLP [79] and ITS [80], [87], where isolates of these two locations grouped separately.

The distribution of isolates according to the geographic origin revealed six transcripts with unique loci that discriminated between geographic clusters: one chitinase, two cellulases, an expansin, a RING and a SKP1. Although the SNPs within the same gene may be segregating together, it may be possible to develop some of these SNPs into molecular markers, providing a unique signature for *B. xylophilus* geographic origin. One interesting gene could be chitinase c9244, which discriminated between the isolates from Portugal, China and Japan, or the SKP1 gene that differentiated the American and the Japanese isolates. Although the polymorphisms detected could be related to a geographic pattern, most of the polymorphic positions were silent, and the few that introduced changes in the amino acid composition were not expected to modify protein function, precluding the inference of different functional profiles related to the geographic origin.

## Conclusions

This study provides the first SNP screening of PWN collected from different geographic locations and demonstrates the power of high throughput transcriptome sequencing to uncover genetic diversity in non-model organisms. The SNPs detected in this study enlarge the number of markers available to address phylogeny and evolution of these parasitic nematodes. Furthermore, the markers provide a means to trace the dispersal and introduction pathways of *B. xylophilus*.

## Supporting Information

File S1 **Supporting Tables S1–S6. Table S1.** Additional *Bursaphelenchus xylophilus* isolates included in the validation of single nucleotide polymorphisms (SNPs) in genomic DNA (gDNA). The validation of SNPs involved 15 *B. xylophilus* isolates, the seven pyrosequenced isolates (Table 1) and eight additional isolates from Madeira Island, China, Japan, USA and a new geographic location, South Korea. **Table S2**. Primer sequences designed for the amplification of *Bursaphelenchus xylophilus* genes for single nucleotide polymorphism (SNP) validation. Primers were designed according to the sequence of the nematode transcripts and used to amplify the corresponding regions in genomic DNA in the 15 *B. xylophilus* isolates. The amplicons were Sanger sequenced to validate SNPs identified in transcripts. **Table S3**. Heterozygotic positions in Japanese (BxJT4) and American (BxUSA618, BxUSA745) *Bursaphelenchus xylophilus* isolates. **Y** for C or T; W for A or T; K for G or T; R for A or G; S for C or G and M for A or C. **Table S4**. Major and minor allele frequencies of the 136 single nucleotide polymorphisms (SNPs) in the 15 *Bursaphelenchus xylophilus* isolates. The allele frequencies were determined by SNPAnalyzer. **Table S5**. Non-synonymous single nucleotide polymorphisms (SNPs) in *Bursaphelenchus xylophilus* isolates resulting in amino acid changes. T – threonine, M – methionine, V- valine, I – isoleucine, G – glycine, S – serine, N – asparagin and E –glutamic acid. **Table S6**. Polymorphic positions exclusive of *Bursaphelenchus xylophilus* isolates from different geographic origins.Single nucleotide polymorphisms (SNPs) were identified in homozigoty in the *B. xylophilus* isolates from specific geographic locations, in one or more transcripts.
(DOC)
