# Supplementary material for: Assessment of the Geographic Origins of Pinewood Nematode Isolates via Single Nucleotide Polymorphism in Effector Genes
Source: PLoS One. 2013 Dec 31;8(12):e83542. doi: 10.1371/journal.pone.0083542 (PMC3877046; doi:10.1371/journal.pone.0083542)
Supplement: File S1 — Supporting Tables S1–S6. Table S1. Additional Bursaphelenchus xylophilus isolates included in the validation of single nucleotide polymorphisms (SNPs) in genomic DNA (gDNA). The validation of SNPs involved 15 B. xylophilus isolates, the seven pyrosequenced isolates (Table 1) and eight additional isolates from Madeira Island, China, Japan, USA and a new geographic location, South Korea. Table S2. Primer sequences designed for the amplification of Bursaphelenchus xylophilus genes for single nucleotide polymorphism (SNP) validation. Primers were designed according to the sequence of the nematode transcripts and used to amplify the corresponding regions in genomic DNA in the 15 B. xylophilus isolates. The amplicons were Sanger sequenced to validate SNPs identified in transcripts. Table S3. Heterozygotic positions in Japanese (BxJT4) and American (BxUSA618, BxUSA745) Bursaphelenchus xylophilus isolates. Y for C or T; W for A or T; K for G or T; R for A or G; S for C or G and M for A or C. Table S4. Major and minor allele frequencies of the 136 single nucleotide polymorphisms (SNPs) in the 15 Bursaphelenchus xylophilus isolates. The allele frequencies were determined by SNPAnalyzer. Table S5. Non-synonymous single nucleotide polymorphisms (SNPs) in Bursaphelenchus xylophilus isolates resulting in amino acid changes. T – threonine, M – methionine, V- valine, I – isoleucine, G – glycine, S – serine, N – asparagin and E –glutamic acid. Table S6. Polymorphic positions exclusive of Bursaphelenchus xylophilus isolates from different geographic origins.Single nucleotide polymorphisms (SNPs) were identified in homozigoty in the B. xylophilus isolates from specific geographic locations, in one or more transcripts. (DOC) [file pone.0083542.s001.doc]

**Assessment of the geographic origins of pinewood nematode isolates via single nucleotide polymorphism in effector genes**

Figueiredo *et al.*

**Supplementary tables**

**Table S1.** Additional *Bursaphelenchus xylophilus* isolates included in the validation of single nucleotide polymorphisms (SNPs) in genomic DNA (gDNA). The validation of SNPs involved 15 *B. xylophilus* isolates, the seven pyrosequenced isolates (Table 1) and eight additional isolates from Madeira Island, China, Japan, USA and a new geographic location, South Korea.

| **Isolate code** | **Geographic origin** | ***Pinus species*** |
| --- | --- | --- |
| BxMad4SV | São Vicente (Madeira Island, Portugal) | *P. pinaster* |
| BxMad16S | Santana (Madeira Island, Portugal) | *P. pinaster* |
| BxChSD | China (Shandong province) | Unknown |
| BxJT4 | Japan (Unknown) | Unknown |
| BxJS10 | Japan (Unknown | Unknown |
| BxUSA745 | USA (Unknown) | Unknown |
| BxKAS | South Korea (Sacheon province) | *P. densiflora* |
| BxKBG | South Korea (Goseung province) | *P. densiflora* |

**Table S2**. Primer sequences designed for the amplification of *Bursaphelenchus xylophilus* genes for single nucleotide polymorphism (SNP) validation. Primers were designed according to the sequence of the nematode transcripts and used to amplify the corresponding regions in genomic DNA in the 15 *B. xylophilus* isolates. The amplicons were Sanger sequenced to validate SNPs identified in transcripts.

| **Gene** | **Transcript name** | **Primer sequences** | **Amplicon Size**  **(bp)** |
| --- | --- | --- | --- |
| **Cellulase** | c4171 | For 5’-CTCCTGGGTTGTCGGAGTTTCC-3’  Rev 5’- CATCTGCGGCGTCAACGACTA-3’ | 615 |
|  | c7206 | For 5’-CCCAAATACCAATCCCAGC-3’  Rev 5’-TGGAGATGCTAACGCCACAA-3’ | 544 |
| **Chitinase** | c1471 | For 5’-TCGGATGCCTTTGCCAAATC-3’  Rev 5’-TGTCCGGCTGGTCAACCATAT-3’ | 778 |
|  | c4666 | For 5’-ATCTGACGAACTGGGCACAAT-3’  Rev 5’-CTCCTTCTTGCACACGTTCTCA-3’ | 876 |
|  | c9244 | For 5’-ATCTGACGAACTGGGCACAAT-3’  Rev 5’-CTCCTTCTTGCACACGTTCTCA-3’ | 629 |
| **Expansin** | c5646 | For 5’-ATGAACTCCCTCTACTTGTTG-3’  Rev 5’-AGCTGATCTTGGTCTGACTG-3’ | 386 |
| **Pectate lyase** | c5837 | For 5’-CCAAAGAAGATGGTGATTAAAG-3’  Rev 5’-AGGCCACTCCTTTTGGCTC-3’ | 484 |
| **SKP1** | c4717 | For 5’-TCCACCTACGTTACTAACCATG  Rev 5’-GACATTTTCGCGTCTGATAC-3’ | 538 |
| **RING** | c2443 | For 5’-TGTTCTTCTCTGCCTGCTGT-3’  Rev 5’-CTGAGGAGCCTGTAGTCTTC-3’ | 685 |
|  | c6704 | For 5’-CAAGTATGGATACCTCCGAGTC-3’  Rev 5’-CCCAATCCTGTTGACAAAGC-3’ | 223 |

**Table S3.**

Heterozygotic positions in Japanese (BxJT4) and American (BxUSA618, BxUSA745) *Bursaphelenchus xylophilus* isolates. **Y** for C or T; **W** for A or T; **K** for G or T; **R** for A or G; **S** for C or G and **M** for A or C.

| **Locus** | **Allele** | **Major allele frequency** | **Minor allele frequency** | | |
| --- | --- | --- | --- | --- | --- |
| Loc1 | C/T | 0.867 | 0,133 |  |  |
| Loc2 | A/G | 0.733 | 0,267 |  |  |
| Loc3 | G/C | 0.733 | 0,267 |  |  |
| Loc4 | T/C | 0.867 | 0,133 |  |  |
| Loc5 | A/C | 0.733 | 0,267 |  |  |
| Loc6 | G/T | 0.733 | 0,267 |  |  |
| Loc7 | C/T | 0.867 | 0,133 |  |  |
| Loc8 | A/C | 0.867 | 0,133 |  |  |
| Loc9 | C/T | 0.733 | 0,267 |  |  |
| Loc10 | T/C | 0.800 | 0,2 |  |  |
| Loc11 | C/T | 0.733 | 0,267 |  |  |
| Loc12 | T/C | 0.800 | 0,2 |  |  |
| Loc13 | C/T | 0.733 | 0,267 |  |  |
| Loc14 | G/A | 0.733 | 0,267 |  |  |
| Loc15 | C/T | 0.800 | 0,2 |  |  |
| Loc16 | T/C | 0.733 | 0,267 |  |  |
| Loc17 | G/A | 0.933 | 0,067 |  |  |
| Loc18 | T/C | 0.800 | 0,2 |  |  |
| Loc19 | G/A | 0.867 | 0,133 |  |  |
| Loc20 | G/A | 0.867 | 0,133 |  |  |
| Loc21 | A/C | 0.867 | 0,133 |  |  |
| Loc22 | C/T | 0.833 | 0,167 |  |  |
| Loc23 | C/T | 0.733 | 0,267 |  |  |
| Loc24 | T/C | 0.733 | 0,267 |  |  |
| Loc25 | G/C/A/T | 0.800 | 0,133 | 0.033 | 0.033 |
| Loc26 | T/C | 0.800 | 0,2 |  |  |
| Loc27 | G/T | 0.800 | 0,2 |  |  |
| Loc28 | G/A | 0.800 | 0,2 |  |  |
| Loc29 | T/C | 0.800 | 0,2 |  |  |
| Loc30 | A/C | 0.800 | 0,2 |  |  |
| Loc31 | T/C/G | 0.767 | 0,2 | 0.033 |  |
| Loc32 | A/T | 0.733 | 0,267 |  |  |
| Loc33 | A/G | 0.767 | 0,233 |  |  |
| Loc34 | G/A/T | 0.800 | 0,133 | 0.067 |  |
| Loc35 | A/G | 0.667 | 0,333 |  |  |
| Loc36 | T/C | 0.833 | 0,167 |  |  |
| Loc37 | T/C | 0.867 | 0,133 |  |  |
| Loc38 | A/G | 0.533 | 0,467 |  |  |
| Loc39 | A/G | 0.667 | 0,333 |  |  |
| Loc40 | A/G | 0.667 | 0,333 |  |  |
| Loc41 | C/G | 0.533 | 0,467 |  |  |
| Loc42 | T/C | 0.667 | 0,333 |  |  |
| Loc43 | A/C | 0.533 | 0,467 |  |  |
| Loc44 | T/A | 0.533 | 0,467 |  |  |
| Loc45 | G/A | 0.800 | 0,2 |  |  |
| **Marker_ID** | **Allele** | **Major allele frequency** | **Minor allele frequency** | | |
| Loc46 | C/G | 0.933 | 0,067 |  |  |
| Loc47 | C/A | 0.600 | 0,4 |  |  |
| Loc48 | A/G | 0.600 | 0,4 |  |  |
| Loc49 | T/C | 0.633 | 0,367 |  |  |
| Loc50 | G/A | 0.633 | 0,367 |  |  |
| Loc51 | G/A | 0.533 | 0,467 |  |  |
| Loc52 | G/A | 0.533 | 0,467 |  |  |
| Loc53 | A/G | 0.533 | 0,467 |  |  |
| Loc54 | C/G | 0.567 | 0,433 |  |  |
| Loc55 | T/C | 0.933 | 0,067 |  |  |
| Loc56 | C//M | 0.897 | 0,069 | 0.034 |  |
| Loc57 | A/G | 0.667 | 0,333 |  |  |
| Loc58 | C/A | 0.667 | 0,333 |  |  |
| Loc59 | T/C | 0.900 | 0,1 |  |  |
| Loc60 | G/C | 0.700 | 0,3 |  |  |
| Loc61 | T/C | 0.767 | 0,233 |  |  |
| Loc62 | G/A | 0.933 | 0,067 |  |  |
| Loc63 | A/G | 0.867 | 0,133 |  |  |
| Loc64 | T/C | 0.900 | 0,1 |  |  |
| Loc65 | G/C | 0.867 | 0,133 |  |  |
| Loc66 | C/G | 0.967 | 0,033 |  |  |
| Loc67 | T/C | 0.733 | 0,267 |  |  |
| Loc68 | C/A | 0.867 | 0,133 |  |  |
| Loc69 | T/C | 0.867 | 0,133 |  |  |
| Loc70 | T/C | 0.867 | 0,133 |  |  |
| Loc71 | C/T | 0.767 | 0,233 |  |  |
| Loc72 | T/C | 0.967 | 0,033 |  |  |
| Loc73 | C/T | 0.800 | 0,2 |  |  |
| Loc74 | T/C | 0.800 | 0,2 |  |  |
| Loc75 | T/C | 0.867 | 0,133 |  |  |
| Loc76 | T/C | 0.867 | 0,133 |  |  |
| Loc77 | C/T | 0.800 | 0,2 |  |  |
| Loc78 | T/C | 0.767 | 0,233 |  |  |
| Loc79 | G/C | 0.867 | 0,133 |  |  |
| Loc80 | C/T | 0.933 | 0,067 |  |  |
| Loc81 | T/C | 0.767 | 0,233 |  |  |
| Loc82 | G/A | 0.900 | 0,1 |  |  |
| Loc83 | A/G | 0.933 | 0,067 |  |  |
| Loc84 | A/C | 0.933 | 0,067 |  |  |
| Loc85 | T/C | 0.933 | 0,067 |  |  |
| Loc86 | A/G | 0.733 | 0,267 |  |  |
| Loc87 | A/G | 0.733 | 0,267 |  |  |
| Loc88 | C/T | 0.933 | 0,067 |  |  |
| Loc89 | A/C | 0.733 | 0,267 |  |  |
| Loc90 | C/T | 0.933 | 0,067 |  |  |
| Loc91 | C/T | 0.733 | 0,267 |  |  |
| Loc92 | T/C | 0.867 | 0,133 |  |  |
| Loc93 | G/T/A | 0.900 | 0,067 | 0.033 |  |
| Loc94 | G/T | 0.733 | 0,267 |  |  |
| Loc95 | A/C | 0.867 | 0,133 |  |  |
|  |  |  |  | | |
| **Marker_ID** | **Allele** | **Major allele frequency** | **Minor allele frequency** | | |
| Loc96 | C/T | 0.933 | 0,067 |  |  |
| Loc97 | G/A | 0.833 | 0,167 |  |  |
| Loc98 | C/T | 0.933 | 0,067 |  |  |
| Loc99 | C/T/A | 0.800 | 0,167 | 0.033 |  |
| Loc100 | T/C | 0.900 | 0,1 |  |  |
| Loc101 | G/C | 0.667 | 0,333 |  |  |
| Loc102 | C/T/G | 0.900 | 0,067 | 0.033 |  |
| Loc103 | A/C | 0.667 | 0,333 |  |  |
| Loc104 | T/C | 0.667 | 0,333 |  |  |
| Loc105 | C/T | 0.800 | 0,2 |  |  |
| Loc106 | C/T | 0.933 | 0,067 |  |  |
| Loc107 | C/T | 0.833 | 0,167 |  |  |
| Loc108 | G/C | 0.833 | 0,167 |  |  |
| Loc109 | C/T | 0.767 | 0,233 |  |  |
| Loc110 | A/G | 0.767 | 0,233 |  |  |
| Loc111 | G/C | 0.933 | 0,067 |  |  |
| Loc112 | T/C | 0.667 | 0,333 |  |  |
| Loc113 | C/T | 0.867 | 0,133 |  |  |
| Loc114 | C/T | 0.800 | 0,2 |  |  |
| Loc115 | C/T | 0.700 | 0,3 |  |  |
| Loc116 | T/C | 0.700 | 0,3 |  |  |
| Loc117 | T/C | 0.900 | 0,1 |  |  |
| Loc118 | C/T | 0.567 | 0,433 |  |  |
| Loc119 | C/T | 0.567 | 0,433 |  |  |
| Loc120 | A/G | 0.867 | 0,133 |  |  |
| Loc121 | C/T | 0.833 | 0,167 |  |  |
| Loc122 | A/G | 0.733 | 0,267 |  |  |
| Loc123 | C/G/T | 0.733 | 0,2 | 0.067 |  |
| Loc124 | A/G | 0.800 | 0,2 |  |  |
| Loc125 | C/T | 0.833 | 0,167 |  |  |
| Loc126 | C/A/G | 0.800 | 0,133 | 0.067 |  |
| Loc127 | G/A | 0.867 | 0,133 |  |  |
| Loc128 | G/A | 0.867 | 0,133 |  |  |
| Loc129 | G/A | 0.867 | 0,133 |  |  |
| Loc130 | T/C | 0.867 | 0,133 |  |  |
| Loc131 | A/G | 0.867 | 0,133 |  |  |
| Loc132 | T/A | 0.867 | 0,133 |  |  |
| Loc133 | G/C | 0.867 | 0,133 |  |  |
| Loc134 | G/A | 0.867 | 0,133 |  |  |
| Loc135 | C/T | 0.933 | 0,067 |  |  |
| Loc136 | A/G | 0.933 | 0,067 |  |  |

**Table S5**. Non-synonymous single nucleotide polymorphisms (SNPs) in *Bursaphelenchus xylophilus* isolates resulting in amino acid changes. T – threonine, M – methionine, V- valine, I – isoleucine, G – glycine, S – serine, N – asparagin and E –glutamic acid.

| **Isolate code** | **SKP1**  **c4717** | | **Chitinase c9244** | **RING c2443** | |
| --- | --- | --- | --- | --- | --- |
| **Locus** | Loc97 | Loc100 | Loc45 | Loc122/123 | Loc129 |
| BxPt15SC | T | V | G | N | V |
| BxPt17AS | T | V | G | N | V |
| BxPt19SCD | T | V | G | N | V |
| BxPt21T | T | V | G | N | V |
| BxMad4SV | T | V | G | N | V |
| BxMad16S | T | V | G | N | V |
| BxChJS | T | V | G | E | M |
| BxChSD | T | V | G | E | M |
| BxJ10 | M | I | S | N | V |
| BxJT4 | T | V/I | S | N | V |
| BxJS10 | M | I | S | N | V |
| BxUSA618 | T | V | G | E | V |
| BxUSA745 | T | V | G | E | V |
| BxKAS | T | V | G | N | V |
| BxKBG | T | V | G | N | V |

**Table S6**. Polymorphic positions exclusive of *Bursaphelenchus xylophilus* isolates from different geographic origins.Single nucleotide polymorphisms (SNPs) were identified in homozigoty in the *B. xylophilus* isolates from specific geographic locations, in one or more transcripts.

| **Isolates origin** | **Transcript** | **Locus** | **Alteration** | **Alteration in other isolates** |
| --- | --- | --- | --- | --- |
| USA | Cellulase c4171 | Loc1 | T | C |
|  |  | Loc4 | C | T |
|  | SKP1 c4717 | Loc110 | T | C |
| Japan | Cellulase c7206 | Loc26 | C | T |
|  |  | Loc27 | T | G |
|  |  | Loc28 | A | G |
|  |  | Loc29 | C | T |
|  |  | Loc30 | C | A |
|  | Chitinase c9244 | Loc45 | A | G |
|  | SKP1 c4717 | Loc99 | T | C |
| Portugal/Korea | Chitinase c9244 | Loc38 | A | G |
|  |  | Loc41 | C | G |
|  |  | Loc43 | A | C |
|  |  | Loc44 | T | A |
|  |  | Loc51 | G | A |
|  |  | Loc52 | G | A |
|  |  | Loc53 | A | G |
| China | Chitinase c9244 | Loc37 | C | T |
|  | Expansin c5646 | Loc92 | C | T |
|  | RING c2443 | Loc120 | A | C |
|  |  | Loc127 | A | G |
|  |  | Loc128 | A | G |
|  |  | Loc129 | A | G |
|  |  | Loc130 | C | T |
|  |  | Loc131 | G | A |
|  |  | Loc132 | A | T |
|  |  | Loc133 | C | G |
|  |  | Loc134 | A | G |
